# Coping with uncertainty in everyday situations (CUES©) to address intolerance of uncertainty in autistic children: an intervention feasibility trial

**DOI:** 10.1007/s10803-022-05645-5

**Published:** 2022-07-05

**Authors:** Jacqui Rodgers, Jane Goodwin, Deborah Garland, Victoria Grahame, Lucy Isard, Ashleigh Kernohan, Marie Labus, Mr Malcolm Osborne, Jeremy R Parr, Priyanka Rob, Catharine Wright, Mark Freeston

**Affiliations:** 1 Population Health Sciences Institute, Sir James Spence Institute, Royal Victoria Infirmary, Newcastle University, Queen Victoria Road, NE1 4LP Newcastle, UK; 2 Complex Neurodevelopmental Disorders Service, Cumbria, Tyne and Wear NHS Foundation Trust, Walkergate Park, NE6 4QD Cumbria, Northumberland UK; 3 Complex Neurodevelopmental Disorders Service, Northumberland, Tyne and Wear NHS Foundation Trust, Walkergate Park, NE6 4QD Cumbria, UK; 4 School of Psychology, Dame Margaret Barbour Building, Newcastle University, NE1 4LP Newcastle, UK; 5 Institute of Health & Society Newcastle University, Baddiley-Clark Building, Richardson Road, NE2 4AX Newcastle upon Tyne, UK; 6 Research and Enterprise Services, Faculty of Medical Sciences, The Medical School, Newcastle University, Framlington Place, NE2 4HH Newcastle upon Tyne, UK; 7 South Tyneside’s Kids And Young Adults Klub - Special needs support group (KAYAKS), NE33 4UG South Shields, Tyne & Wear, UK; 8 Child and Adolescent Mental Health Service, Northumbria Healthcare NHS Foundation Trust, Albion Rd Resource Centre, NE29 0HG North Shields, UK; 9 School of Psychology, Newcastle University, 4th Floor Ridley Building, NE1 7RU Newcastle upon Tyne, Tyne and Wear, UK

**Keywords:** Anxiety, Intolerance of uncertainty, Parent group, Intervention, Treatment, Autism, ASD

## Abstract

**Background:**

Anxiety related to uncertainty is common in autism. Coping with Uncertainty in Everyday Situations (CUES©) is a parent-mediated group intervention aiming to increase autistic children’s tolerance to uncertain situations. A pilot study was conducted to test its feasibility and acceptability.

**Methods:**

Parents of 50 autistic children were randomised to receive CUES© or enhanced services as usual.

**Results:**

All children met the clinical threshold for at least one anxiety disorder. Of the 26 participants randomised to CUES©, 72% attended 4–8 sessions. Parents and therapists reported they found CUES© useful and acceptable.

**Conclusions:**

Families were willing to be recruited and randomised, the format/content was feasible to deliver, and the outcome measures were acceptable. CUES© should be evaluated in a clinical and cost effectiveness randomised controlled trial.

Almost 50% of autistic children experience clinical levels of anxiety (Simonoff et al., 2008; van Steensel et al., 2011). Anxiety can negatively impact an autistic child’s participation and enjoyment, academic performance, and interactions with others (Adams & Emerson, 2020). Anxiety is an autism research priority (Autistica, 2016). Autistic individuals frequently present with features from multiple anxiety disorders concurrently (Rodgers et al., 2017) as well as autism related anxiety symptoms (Kerns et al., 2014). Therefore, treatments targeting underlying anxiety mechanisms may be most efficacious (Wilkinson et al., 2011).

Difficulties coping with uncertain situations (known as intolerance of uncertainty, hereafter IU) has been identified as a key transdiagnostic mechanism involved in anxiety (Carleton et al., 2012). IU involves the ‘tendency to react negatively on an emotional, cognitive and behavioural level to uncertain situations and events’ (Buhr & Dugas, 2009). In non-autistic populations, treatments focusing on IU are reported to be effective (Hebert & Dugas, 2019; Wahlund et al., 2020). Also, IU is an important aspect of anxiety for autistic people (Sáez-Suanes et al., 2020). A recent meta-analysis found a strong relationship between anxiety and IU within autistic populations (Jenkinson et al., 2020).

We have developed a programme to address difficulties related to uncertain situations for autistic children. Coping with Uncertainty in Everyday Situations (CUES©; Rodgers et al., 2019; Rodgers et al., 2017) was developed in collaboration with parents; and professionals and is a parent group intervention to enable parents to support their child in everyday uncertain situations.

We aimed to investigate the acceptability and feasibility of recruiting to a randomised controlled trial through the UK National Health Service (NHS), delivering CUES©, and measuring outcomes, and had the following specific goals:

Our feasibility goals are:

- To explore whether CUES© is feasible to be delivered via NHS clinicians through feedback from therapists.
- To investigate whether treatment fidelity can be maintained across multiple trained therapists.

Our acceptability goals are:

- To determine the number of parents who agree to participate in the study.
- To determine the average number of sessions attended by parents in the CUES and Understanding Autism attentional conditions.
- To determine the attrition rate in the CUES and Understanding Autism attentional control conditions.
- To explore the completion rates of outcome measures at baseline and follow ups.
- To explore whether CUES© is acceptable to families and explore the acceptability of our outcome measures through feedback from parents.

Whilst acknowledging that the study is not a fully powered trial a final goal was to undertake some preliminary evaluation of treatment affects at 6 months following intervention.

## Methods

The methods have been published previously (Rodgers et al., 2019) and are summarised below.

## Participants

Inclusion criteria: Parents of a child aged 6–16 years with a confirmed clinical autism diagnosis. All of the children were currently accessing clinical services due to anxiety. Referring clinicians were provided with information about the study, including documentation about how IU presents for autistic children. The referring clinicians were asked to consider families on their caseload and identify, using clinical judgement, any children who were experiencing anxiety related to uncertainty. They were asked to discuss the study with parents and provide them with information about the study and an expression of interest form to complete if they were interested in taking part. Parents who completed the expression of interest form were then contacted by the research team to determine eligibility. In order to be eligible, children had to meet clinical cut off for at least one anxiety disorder on the ADIS and the parent was required to be able to identify two everyday uncertain situations that caused their child some difficulty during the Uncertain Situations Interview. No assessment of child intellectual ability was undertaken as part of the study and parents of children with a diagnosis of mild intellectual disability were also invited to take part if they met the inclusion criteria specified above. Parents with sufficient English to complete outcome measures.

Exclusion criteria: Parents of children with a severe and complex anxiety disorder (based on clinicians’ clinical judgement), severe intellectual disability, complex health condition.

Four hundred and twenty-four study packs were given to NHS multidisciplinary autism diagnostic and mental health teams. The number of packs handed to parents is unknown. Eighty families expressed interest in participating. Of these, 28 were excluded (one declined to participate, 25 could not be contacted, one sent an expression of interest form after recruitment had closed) and 2 withdrew after consent. Fifty-one families completed baseline assessments and progressed to randomisation. One family withdrew following randomisation, leaving 50 families in the study.

## Measures

### Baseline characterisation

#### Social Communication Questionnaire (SCQ; Rutter et al., 2003)

This 40-item questionnaire assessed child characteristics and functional abilities.

#### Vineland adaptive Behaviour Scales 3 (VABS 3; Sparrow et al., 2016)

With this measure the parent/caregiver rates aspects of their child’s level of adaptive functioning focused on four domains: communication, daily living skills, socialisation and motor skills. These subscales are combined into an Adaptive Behavior Composite, which provides an overall summary measure of the individual’s adaptive functioning.

#### Anxiety Disorders interview schedule—autism Spectrum Addendum (ADIS-ASA; Kerns, Silverman, & Albano, in press)

The ADIS assesses child anxiety. The subscales include DSM anxiety diagnoses (Generalised Anxiety Disorder, Specific Phobia, Social Phobia, Separation Anxiety Disorder, Panic Disorder, Agoraphobia, and Other Anxiety disorder); ADIS-ASA specified conditions (Fear of Change or Negative Reaction to Change, Idiosyncratic Phobia, Other Social Fear, and Special Interest Fear); and other specified conditions (Post-Traumatic Stress Disorder and Obsessive Compulsive Disorder). Trained research psychologists interviewed parents about the impact of anxiety symptoms on the child’s life (range from 0 to 8, where 0 = no interference in child’s life and 8 = very, very much (debilitating fear where hospitalisation should be considered). Scores **≥** 4 indicate the clinical threshold for an anxiety disorder diagnosis.

### Primary outcome measures

#### Parent interview

A bespoke parent interview determined the acceptability of all aspects of the trial (e.g., acceptability of intervention), and feasibility (e.g., experience of recruitment).

#### Credibility and expectancy questionnaire (CEQ; Devilly & Borkovec 2000)

The CUES© group completed a 10-item questionnaire and the Understanding Autism group completed a 6-item questionnaire about feasibility and acceptability. The subscales are cognitively based credibility and affectively based expectancy.

#### Therapist interview

A bespoke interview explored therapists’ experiences of delivering CUES©, including acceptability and feasibility of training, materials, pacing, and engagement.

### Secondary outcome measures

#### Screen for child anxiety related Disorders (SCARED; Birmaher et al., 1997)

This 41-item questionnaire assesses child anxiety and has good internal consistency (α = 0.74–0.93), test-retest reliability (ICC 0.70–0.90), and discriminative validity (Birmaher et al., 1997). The subscales are Generalised Anxiety Disorder, Social Anxiety Disorder, Separation Anxiety Disorder, Panic Disorder, and Significant School Avoidance.

#### Intolerance of Uncertainty scale (IUS-P and IUS-C; Walker 2009)

The parent and child Intolerance of Uncertainty Scales provide parent and child self-report of child IU using a 12-item questionnaire. Respondents rate, on a five-point Likert scale, whether statements relating to emotional, cognitive and behavioural responses to IU relate to the child.

#### Anxiety Scale for children — ASD – parent and child versions (ASC-ASD; Rodgers et al., 2016)

The ASC ASD provides a measure of child anxiety. Both parent and child versions have 24 items, good to excellent internal consistency (α = 0.85–0.91), validity with other measures (e.g., depression and anxiety), and 1 month test–retest reliability (*r* = 0.84, ICC = 0.84 for parent; *r* = 0.82, ICC = 0.82 for child) (Rodgers et al., 2016). The subscales are Uncertainty, Performance Anxiety, Separation Anxiety, and Anxious Arousal.

### Bespoke questionnaires

Bespoke questionnaires were developed to assess (a) demographics; (b) health resource use associated with child IU; (c) time and travel costs associated with managing child IU; (d) reactions to uncertainty and confidence (administered each week of the CUES© programme).

### Target Uncertain Situation interview

Parents identified two target real-life situations causing their child significant IU (method previously validated; Rodgers et al., 2017) via a standardised interview: one situation their child would like to do but currently cannot do or cannot do consistently (e.g. playing with friends in the neighbourhood); and another situation that is appropriate and necessary due to typical developmentally appropriate educational, social participation, or inclusion outcomes (e.g. swimming lessons). Parents were interviewed about the situation, what was uncertain, frequency, the child’s reaction, whether the child worries about the situation in advance, and interference with daily functions and activities for the child and family.

This assessment protocol is based on that used by The Research Unit on Paediatric Psychopharmacology and Psychosocial Interventions (Arnold et al., 2003) and was used in previous treatment trials (e.g., Maskey et al., 2019). The IU target situations were rated on a nine-point scale of improvement/deterioration independently by a panel of experienced clinicians. The top 3 points define a ‘responder’.

### Intolerance of uncertainty scale (IUS-12; Carleton et al., 2012)

The IUS 12 provides a rating of parent IU on a five-point Likert scale, and includes 12 statements about their emotional, cognitive and behavioural responses to IU.

### Depression, anxiety and stress scale (DASS; Lovibond & Lovibond 1995)

This 42-item questionnaire was used to assess parent wellbeing. It has acceptable reliability (α = 0.84–0.91), and convergent and discriminant validity (Lovibond & Lovibond, 1995). The subscales are Depression, Anxiety, and Stress.

### Parent self-efficacy (Sofronoff & Farbotko, 2002)

Parents rated their confidence and self-efficacy in relation to 15 behaviours targeted in the study.

### Children’s Assessment of participation and enjoyment (CAPE; King et al., 2004)

This 55-item questionnaire assessed participation and enjoyment. It has good internal consistency (α = 0.30–0.62, expected due to factors that affect participation), test–retest reliability (ICC = 0.64–0.86), and sufficient content and construct validity (King et al., 2004). The subscales are Diversity, Enjoyment, Where, With Whom, and Intensity (Table 1).

**Table 1:** Time points at which data is collected

Measures	Baseline	During Treatment	Immediately post-treatment	6 weeks post-treatment	12 weeks post-treatment	26 weeks post-treatment
**Baseline characterisation**						
SCQ^a^	X					
VABS 3^a^	X					
ADIS-ASA^a^	X					
**Primary outcome measures**						
Parent interview^b^				X		
Credibility and Expectancy questionnaire^c^		X (CUES sessions 2, 5, 8; Understanding Autism session 1)				
Therapist interview^d^				X		
**Secondary outcome measures**						
SCARED^a^	X		X		X	X
IUS-P^a^	X		X		X	X
IUS-C^e^	X		X		X	X
ASC-ASD Parent version^a^	X		X		X	X
ASC-ASD Child version^e^	X		X		X	X
Bespoke questionnaires: demographic information, time and travel information, resource use^b^	X					
Target Uncertain Situation^a^	X		X		X	X
IUS-12^c^	X		X		X	X
DASS^c^	X		X		X	X
Parent self-efficacy^c^	X		X		X	X
CAPE^e^	X		X		X	X
Reactions to uncertainty and confidence^b^		X (all CUES sessions)				

^a^ Indicates that the parent was reporting on their child

^b^ Indicates that the parent was reporting on themselves and their child

^c^ Indicates that it was parent self-report

^d^ Indicates that it was therapist self-report

^e^ Indicates that the measure was completed by the child (with help, if needed)

## Procedure

A favourable ethical opinion was provided by Tyne and Wear South NHS Research Ethics Committee on 17 April 2018, ref. 18/NE/0106  and was conducted according to the World Medical Association Declaration of Helsinki.Sponsorship was provided by Cumbria, Tyne and Wear NHS Foundation Trust.

Clinicians from UK NHS services (autism diagnostic clinics and Child and Adolescent Mental Health Services) identified families with children meeting inclusion criteria. Clinicians discussed the study with families and gave study packs to those interested. Families returned expression of interest forms to the research team, who telephoned the families to discuss the study and arrange a home visit to take informed parent consent, child assent, and conduct baseline assessments. Potential participants were informed that this was a study to determine the feasibility and acceptability of an intervention developed to help parents of autistic children support their child to cope with uncertain situations. It was emphasised that regardless of group allocation, there would be helpful information and the opportunity to meet other parents at each group. The research team also explained the importance of attending and completing follow up measures as contributing to the results of the overall study, which may inform future care. Eligible participants were randomised in a 1:1 ratio to ‘CUES©’ or ‘Understanding Autism’, without stratification. Randomisation occurred online through Sealed Envelope (https://www.sealedenvelope.com/).

Four sets of groups ran from October 2018 – November 2019 during school terms. Follow up assessments took place immediately at the end of the group intervention (8 weeks) and at two further time points: 12 and 26 weeks after the intervention. Parent and therapist interviews regarding feasibility and acceptability were conducted 6 weeks after intervention. These were completed via telephone or face-to-face, depending on their preference. All families remained under the clinical responsibility of local teams and continued to receive existing routine care during the study.

## Intervention

### Intervention arm: CUES©

CUES© is an eight-week manualised programme (Rodgers et al., 2017, 2019) for parents of autistic children. The goal is to increase the child’s ability to cope with everyday uncertainty, reduce negative beliefs about uncertainty and develop a more flexible approach to uncertainty. It provides the opportunity for parents to develop an understanding of uncertainty and its impact, try out strategies and shared opportunities for discussion and support.

Each CUES© session lasted  2 hours and was facilitated by two therapists with autism expertise. Parents were provided with weekly intervention materials and individual support to identify and try strategies to address a chosen uncertain situation (identified at baseline). ‘At home’ activities were set each week to complete between weekly sessions. If parents missed a session clinicians posted the intervention materials to parents prior to the next session and telephoned them to enable them to address any queries about the the materials and answer any questions.

### Enhanced services as usual: understanding Autism

Understanding Autism (UA) comprised two parent group sessions. Each session lasted 2 hours and was facilitated by two group leaders with knowledge about autism and involvement in research and community outreach. The materials were developed in collaboration with the research team. UA focused on psychoeducation, social communication, repetitive behaviours, and making and keeping friends. There were opportunities for parents to engage in group discussion.

### Fidelity

Two independent raters were randomly allocated 30% of recorded group intervention sessions to rate for fidelity to the treatment manual, using a checklist developed for the study. Raters assessed fidelity using a three-point scale (0—not at all; 1—briefly covered but insufficiently; 2—covered adequately), and therapeutic best practice rated: 0—not at all; 1—minimal evidence; 2—several examples.

### Data analysis

Patterns of recruitment, retention and participation in the intervention were examined. No formal economic analysis was planned but questionnaires were piloted to aid a future economic evaluation.

Interview data were analysed to explore feasibility and acceptability. The analysis was informed by Thematic Analysis (Braun et al., 2019), which is an open and exploratory design and analytic process, prioritising researcher subjectivity and reflexivity (e.g., Gough & Madill 2012). First, author JG listened and re-listened to the audio recordings. Author JG coded the transcriptions, and the coding was sense checked by authors PR, LI, and JG . We used an inductive approach. That is, we identified meaning from the interviews rather than using pre-determined codes to review and analyse the data. The codes were then combined into categories using thematic analysis. The authors engaged in robust discussion of the themes to resolve minor discrepancies, remain cognisant of positionality, and ensure reflexive practice. Saturation was reached for both parent and therapist participants.

Comparisons of the treatment groups was based on the whole randomised sample using an intention to treat analysis, to provide a more conservative estimate of difference between the two conditions and to minimise risk of bias. Independent t tests and Chi square tests were used to compare groups on baseline scores.

## Community involvement

Autistic people and their families were involved and informed of all stages of the research. The author team comprises researchers, scholars, clinicians, advocates, and community leaders. The author team also have personal experience as autistic adults, parents and caregivers of autistic children and family members and friends of autistic individuals. Therefore, autistic adults and parents of autistic children were involved in the design and management of the research as co-applicants and members of the advisory group. The advisory group met regularly for consultation regarding specific aspects of the study (e.g., study documentation, language use).

## Results

### Feasibility goals

#### Therapist interviews

To explore whether CUES© is feasible to be delivered via NHS clinicians through feedback from therapists all 8 CUES© therapists completed a semi-structured interview.

### Delivering CUES©

Two main themes emerged: (1) therapists commented on the “positive experience” of delivering the programme because it “all made sense”, “the sessions built on from previous sessions well”, and they were working with “lovely parents”; and (2) the active role of the therapist to guide which uncertain situation to concentrate on and to keep the discussions focused “it’s finding the balance between making people feel heard and keeping them on track”.

### Programme content

Three main themes emerged: (1) clear explanations of IU with examples “the sorting task was good” and the value of repetition “therapists might think of it as repetition but for parents it’s consolidation”; (2) the balance between activities and discussion was helpful for group cohesion “it helped the group to gel and support each other”; and (3) it could be difficult for parents to find time to complete the homework even though they were thinking about the concepts; however, “in the discussion they could still talk through and had thought about it.”

### Size and pace

Three themes were elicited: (1) the programme was a good pace because it “didn’t feel rushed” and “fit into two hours and left room for discussion”; (2) the group could run comfortably with 10 parents as intended but it was nice to have smaller groups “to form relationships with everyone contributing”; and (3) catch up calls were valuable when parents missed sessions “it was good to ring parents to discuss”.

### Looking to the future

Two main themes emerged: (1) therapists perceived that parents finished the programme feeling “positive”, “empowered”, and “more confident” and were sad when it ended; and (2) all therapists commented that they would like to integrate CUES© into their practice,: “I was happy to have the opportunity to run this programme” and “it was giving skills to spot the cause of the child’s distress and tools to help parents’ distress… really worthwhile”.

#### Fidelity of intervention

In line with our stated goal to investigate whether treatment fidelity can be maintained across multiple trained therapists, adherence to the content of the CUES© manual was rated as 97% and delivery of therapeutic best practice as 96%.

### Acceptability goals

To explore whether CUES© was acceptable to families, CUES participants completed the CEQ and session based on Reactions to Uncertainty and Confidence questions. Using the CEQ all CUES© participants reported that the programme helped them to support their child’s development and manage their anxiety more effectively, see Table 2.

**Table 2:** Results from parent completed CUES© Credibility and Expectancy Questionnaires. Response of 0–9 where 9 indicates most improvement/satisfaction

Question	Mean rating		
	**Session 2** n = 20^a^	**Session 5** n = 18	**Session 8** n = 15
**Looking to the future, how**:			
Logical does CUES seem?	7.1	7.7	8.5
Useful do you think CUES will be in helping you to support your child’s development more effectively?	6.8	7.0	7.9
Useful do you think CUES will be in helping you to manage your child’s anxiety more effectively?	6.7	7.3	8.1
Confident would you be in recommending CUES to a friend who has a child with ASD?	7.1	8.3	8.9
Much further improvement in supporting your child’s development do you think will occur?	6.5	6.7	7.0
Much further improvement in managing your child’s anxiety more effectively do you think will occur?	6.7	7.0	7.1
Much do you really feel that CUES will help you to support your child’s development more effectively?	6.4	6.9	7.5
Much do you really feel that CUES will help you to manage your child’s anxiety more effectively?	6.6	7.1	7.5
Much further improvement in supporting your child’s development more effectively do you really feel will occur?	6.3	7.1	7.5
Much further improvement in managing your child’s anxiety more effectively do you really feel will occur?	6.7	7.3	7.5

^a^ Although only 19 people attended the second session (see Table 4), one parent who did not attend the second session returned the questionnaire after receiving the session’s materials in the post and a telephone catch up with the therapists

Table 3 demonstrates that CUES© participants generally felt more confident and found their child’s reactions to uncertainty less challenging as the programme progressed. The child’s negative reactions to uncertainty tended to decrease but their target uncertain situation caused a stronger reaction at session 8 compared to the previous 3 sessions, which may be consistent with parents gradually exposing their child to that situation as the progress progressed.

**Table 3:** Participants’ reports on their perceptions of themselves and their child, administered each week of the CUES© programme. Response of 0–5, where 5 indicates greater confidence (question 1), more challenge (question 2), and stronger reactions (questions 3–4)

Question	Session 1 n = 20	Session 2 n = 20	Session 3 n = 17	Session 4 n = 16	Session 5 n = 16	Session 6 n = 15	Session 7 n = 18	Session 8 n = 14
1. How confident have you felt about managing your child’s reactions to uncertainty over the last week?	3.0	3.2	3.4	3.5	3.6	4.0	4.2	4.5
2. How challenging have you found your child’s reactions to uncertainty in the last week?	4.0	3.8	3.3	3.1	2.8	3.2	2.8	2.5
3. How has your child reacted to uncertainty in general over the last week?	3.8	3.8	3.4	3.4	3.3	3.2	3.1	2.6
4. How has your child reacted to their target uncertain situation over the last week?^**a**^	-	-	-	3.3	2.8	2.7	2.3	3.0

^**a**^Target situation identified in session 3; therefore, this question was not asked until session 4

#### Participation and attrition

Our acceptability goals included determination of the number of parents who agree to participate in the study, the average number of sessions attended by parents in the CUES© and UA conditions, the attrition rate in the CUES© and UA condition and the completion rates of outcome measures at baseline and follow ups.

Of the 26 participants randomised to CUES©, one withdrew without attending any sessions, citing the programme was not suitable for them. Four CUES© participants did not attend sessions (2 had caregiving duties; 2 did not give a reason) or complete any follow ups. Of the remaining 21 participants, two attended the first two sessions only (one due to ill health; one did not give a reason). Both provided follow up data for their target uncertain situations only. One parent completed 3 sessions before withdrawing due to ill health. The other 18 parents in the CUES© group attended 4–8 sessions and missed sporadic sessions due to work commitments, medical appointments, family illness  , and their child being too anxious to attend school. Only one of the 18 regular attendees were lost to follow up. CUES© therapists indicated that the level of participation and attendance is in line with what they would routinely see for comparable programmes in their clinical services.

In the UA group, 12 of the 25 participants did not attend any sessions, and of these, 7 did not provide any follow up data. Of these 12, two did not attend due to work commitments, one declined attendance following randomisation, one did not have childcare (funding was offered to support this), and 8 did not give a reason. Of the 13 who attended the UA sessions, three participants attended the first session but not the second session (two due to ill health; one did not give a reason) and they provided incomplete follow up data. All other parents (n = 10) attended both sessions and completed at least one follow up. No serious adverse events were reported in either the CUES© or UA groups.

**Table 4:** Attendance at CUES© and UA groups by week

Attendance	Session 1	Session 2	Session 3	Session 4	Session 5	Session 6	Session 7	Session 8
CUES©	19	19	17	14	13	16	17	15
UA	13	10	-	-	-	-	-	-

*There were 25 participants allocated to each group

#### Parent interviews

To further explore acceptability goals nineteen CUES© participants completed a semi-structured interview.

### Acceptability of the intervention

Four main themes emerged: (1) most participants did not know of the role of IU in their child’s anxiety before taking part in this study and it “opened up a whole thing, the whole concept was a revelation”; (2) having therapists with specialist, targeted insight into autism and anxiety “was a gift” and “brilliant”; (3) positive experience of sharing with other parents “it was nice to say what you wanted without judgement” and feeling less alone; and (4) many parents described “the long road ahead”: they were now equipped with tools to manage their child’s IU but their child had lots of worries and likely would do as they grew. Each said that the programme was useful and enjoyable, and they would recommend it for all parents.

### Research process

Two main themes were elicited: (1) information about the study and questionnaires were “useful” and “helpful”, and (2) some parents found the questions difficult because they did not realise so many of their child’s behaviours were anxiety and thought they should have done more to support their child. All participants understood the randomisation process and were pleased to be placed in the CUES© group.

### Impact of the intervention on the participants, their children and the family

Three themes emerged: (1) improved ability to identify and manage their child’s IU “I’ve got better understanding of his anxiety” and shared this information with relatives, friends, and colleagues; (2) changes in their child’s response to anxiety provoking situations: “I understand him and it makes a difference to him” and “he’s got more understanding in himself and can pinpoint uncertainty”; and (3) the positive change CUES© had on them personally. They made time for themselves and felt “less stressed and emotional”: “I’ve got a spring in my step and can see a way forward for my child” and “Absolutely, without a shadow of a doubt… it’s changed my life. Thank you.”.

#### Baseline equivalence of groups

The average age of the children was 10.25 years, and there were 34 males. There were no differences between group allocation (Table 5).

**Table 5:** Means and SD for child baseline characterisation

	Baseline		
	CUES©, N = 25* (SD)	UA, N = 25* (SD)	Difference *p*
**Child age in years**	10.8 (2.9)	9.7 (2.2)	0.114
**Child sex**	18 male	16 male	0.544^1^
**Service referred from** Community Regional specialist	18 7	17 8	0.758
**Parent education** Tertiary/further education Secondary education Other general education	18 7 0	16 7 2	0.347^1^
**Parent-completed measures**			
**SCQ** *A score ≥ 15 suggests the child is likely to be on the autism spectrum*	20.7 (5.7) 20 (80)	n = 24 19.8 (6.8) 18 (72)	0.618
**VABS 3 (adaptive behaviour composite)** *Range is 20–140. A score < 100 indicates adaptive functioning level is below children of the same age*	n = 24 66.8 (8.2)	n = 24 68.0 (9.7)	0.621

*N = 25 for both groups unless otherwise indicated. All percentages calculated out of group allocation total; that is, 25 in each group

^1^Pearson’s Chi Square. All other analyses are independent t-tests

The ADIS-ASA demonstrated all children met the clinical threshold for at least one anxiety disorder (Supplementary Materials 1). Supplementary material 2 contains descriptive results for all child self-report measures. Twenty-nine children were willing and able to complete some questionnaires. Five could not complete questionnaires due to age (6–7 years) or were unable to/did not want to (n = 3, n = 13 respectively); but their parents found the measures acceptable. No further analysis was completed on child self-report data.

#### Health economics

Both the resource use and time and travel questionnaires were fully completed by all participants (Table 6). At the next three follow ups the resource questionnaire was completed entirely by 23, 26 and 28 participants respectively. Those who did not complete the resource use questionnaire also did not complete the other questionnaires administered at that time point. Many participants reported visits to the Children and Young People’s Service, suggesting use of autism and mental health services should be specifically asked about. We conclude that the economics questionnaires were acceptable in terms of content but there may be issues with the overall burden of data collection.

**Table 6:** Completion of resource use and time and travel questionnaires

	Baseline	Follow Up 1	Follow Up 2	Follow Up 3
**Number of participants total** **CUES©** **Understanding Autism**	50 25 25	50 25 25	50 25 25	50 25 25
**Completed Questionnaires Overall total** **CUES©** **Understanding Autism**	50	23 (46%) 15 (60%) 8 (32%)	26 (52%) 15 (60%) 11 (44%)	28 (56%) 15 (60%) 13 (52%)
**Completed Economic Questionnaires total** **CUES©** **Understanding Autism**	50	23 (46%) 15 (60%) 8 (32%)	26 (52%) 15 (60%) 11 (44%)	28 (56%) 15 (60%) 13(52%)

### Treatment effects

Participants identified a wide range of uncertain everyday situations that were difficult. The most common ‘had to’ situations were school related, relating to the uncertainty associated with tests/homework, changes to routine/teachers, and getting in trouble. The most common ‘wanted to’ uncertain situations identified were extracurricular activities, because children did not know what to expect, who would be there, and whether they would be able to do what they needed to do (Goodwin et al., 2021 ). See supplementary material 3 (Table 7 and 8).

**Table 7:** Means and SD of parent report on child outcome variables at baseline and primary endpoint (26 weeks;  intention to treat)

Parent report on child					
	**Baseline**		**Baseline equivalence**	**Follow up 3: Primary endpoint, 26 weeks post-treatment**	
	Mean (SD)			Mean (SD)	
	CUES©	UA	*p*	CUES©	UA
**ASC-ASD-P** Total Uncertainty Performance Anxiety Separation Anxiety Anxious Arousal *Score ≥ 20 indicates significant anxiety levels, score > 24 suggests specific anxiety*	n = 24 34.9 (10.8) 14.1 (4.7) 8.3 (4.8) 7.8 (3.2) 4.8 (3.2)	n = 24 35.7 (11.2) 16.4 (4.9) 8.2 (4.7) 7.5 (3.8) 3.6 (3.3)	0.952 0.228 0.838 0.109 0.802	n = 24 32.9 (12.4) 14.2 (6.3) 7.7 (4.9) 6.9 (3.1) 4.2 (3.0)	n = 24 36.4 (12.9) 15.9 (5.4) 8.2 (4.7) 8.1 (4.5) 4.1 (3.5)
**IUS-P** *Range is 12–60, high score indicates IU characteristics apply to the child*	n = 24 45.9 (7.8)	n = 24 47.0 (6.7)	0.595	n = 24 43.8 (9.8)	n = 24 47.4 (6.7)
**SCARED** Total Generalised Anxiety Disorder Social Anxiety Disorder Separation Anxiety Disorder Panic Disorder Significant School Avoidance *Total score ≥ 25 might indicates an Anxiety Disorder, with scores > 30 suggesting a specific Anxiety Disorder*	n = 25 42.1 (12.6) 11.6 (3.9) 9.4 (3.9) 9.0 (2.9) 8.3 (5.7) 3.8 (2.4)	n = 23 39.0 (16.2) 10.0 (5.4) 9.6 (3.2) 7.8 (4.1) 7.2 (5.6) 4.2 (2.5)	0.520 0.234 0.228 0.843 0.517 0.458	n = 25 38.8 (15.1) 11.0 (3.8) 8.8 (4.3) 7.6 (3.5) 7.5 (6.3) 4.0 (2.7)	n = 23 39.6 (16.1) 10.8 (4.9) 10.1 (2.7) 7.9 (4.8) 6.8 (5.6) 3.8 (2.2)

Note: Some data are missing due to incomplete baseline assessments

**Table 8:** Means and SD of parent self-report outcome variables at baseline, and primary endpoint (26 weeks; intention to treat)

Parent self-report					
	**Baseline**		**Baseline equivalence**	**Follow up 3: Primary endpoint, 26 weeks post-treatment**	
	Mean (SD)			Mean (SD)	
	CUES©	UA	*p*	CUES©	UA
**IUS-12** *Range is 12–60, high score indicates IU characteristics apply to the parent*	n = 25 31.3 (8.9)	n = 23 31.7 (11.2)	0.876	n = 25 27.6 (9.9)	n = 23 31.0 (10.6)
**Parent self-efficacy** *0 = no confidence in managing behaviour, 5 = completely confident*	n = 22 3.6 (0.8)	n = 21 3.6 (1.3)	0.764	n = 22 4.0 (1.0)	n = 21 3.5 (1.2)
**DASS** Stress Participants who met ‘severe’ experience threshold Depression Participants who met ‘severe’ experience threshold Anxiety Participants who met ‘severe’ experience threshold *0 = no experience of symptoms, 21+, 15+, and 26 + = severe experience of symptoms respectively*	n = 25 15.8 (10.2) 5.0 (20.0) 10.7 (10.4) 5.0 (20.0) 7.2 (7.1) 3.0 (12.0)	n = 23 19.3 (9.3) 7.0 (28.0) 14.1 (11.3) 6.0 (24) 11.7 (9.9) 7.0 (28.0)	0.288 0.075 0.215	n = 25 14.1 (11.1) 10.7 (11.4) 6.4 (8.4)	n = 23 20.1 (9.8) 14.4 (12.9) 11.8 (10.5)

Note: Some data are missing due to incomplete baseline assessment

There was no difference between groups on their target uncertain situations at follow-ups one and two (see supplemental data). However, the CUES© group included more ‘responders’ for both target symptoms and family impact (n = 12 and 11 respectively) in the ‘want to’ situation compared to the UA group (n = 2 and 2) at the primary endpoint X^2^ (2, N = 29) = 7.156, p = .028 and X^2^ (2, N = 29) = 5.955, p = .051 respectively 9.

**Table 9:** Data received for target uncertain situation follow ups recoded as ‘responder’, ‘no change’, ‘worse than baseline’

	CUES©	UA	Difference *p*	Effect size ^a^ (Cramér’s V)
**Follow up 3: Primary endpoint, 26 weeks post-treatment**				
**Target Symptoms (want to)** Responder No Change Worse than Baseline	n = 18 12 (48) 6 (24) 0 (0)	n = 11 2 (8) 8 (32) 1 (4)	0.028	0.422
**Family Impact (want to)** Responder No Change Worse than Baseline	11 (44) 7 (28) 0 (0)	2 (8) 8 (32) 1 (4)	0.051	0.326
**Target Symptoms (have to)** Responder No Change Worse than Baseline	n = 18 9 (36) 7 (28) 2 (8)	n = 18 4 (16) 7 (28) 0 (0)	0.306	0.211
**Family Impact (have to)** Responder No Change Worse than Baseline	10 (40) 6 (24) 2 (8)	6 (24) 4 (16) 1 (4)	0.977	0.175
**Follow up 2: 12 weeks post-treatment**				
**Target Symptoms (want to)** Responder No Change Worse than Baseline	n = 13 6 (24) 6 (24) 1 (4)	n = 13 2 (8) 9 (36) 2 (8)	0.231	0.284
**Family Impact (want to)** Responder No Change Worse than Baseline	8 (32) 5 (20) 0 (0)	4 (16) 7 (28) 2 (8)	0.160	0.338
**Target Symptoms (have to)** Responder No Change Worse than Baseline	n = 13 6 (24) 6 (24) 1 (4)	n = 12 3 (12) 6 (24) 3 (12)	0.375	0.271
**Family Impact (have to)** Responder No Change Worse than Baseline	6 (24) 6 (24) 1 (4)	2 (8) 8 (32) 2 (8)	0.275	0.274
**Follow up 1: Immediately post-treatment**				
**Target Symptoms (want to)** Responder No Change Worse than Baseline	n = 11 1 (4) 6 (24) 4 (16)	n = 9 0 (0) 5 (20) 4 (16)	0.638	0.143
**Family Impact (want to)** Responder No Change Worse than Baseline	2 (8) 8 (32) 1 (4)	1 (4) 8 (32) 0 (0)	0.564	0.169
**Target Symptoms (have to)** Responder No Change Worse than Baseline	n = 11 1 (4) 7 (28) 3 (12)	n = 9 0 (0) 5 (20) 4 (16)	0.525	0.151
**Family Impact (have to)** Responder No Change Worse than Baseline	1 (4) 9 (36) 1 (4)	0 (0) 6 (24) 3 (12)	0.298	0.201

Note. All percentages calculated out of sample total; that is, 25 in each group

^a^ Results of Cramer’s V coefficient test: >0.25 = very strong, > 0.15 = Strong, > 0.10 = moderate, > 0.05 = weak and > 0 = no or very weak

## Discussion

This is the first pilot randomized controlled trial of a parent group intervention (CUES©) designed to help parents support their autistic child to cope better with everyday uncertain situations. Results support the feasibility and acceptability of CUES© and the recruitment and research procedures. Therapists were able to deliver the programme with a high degree of fidelity to the treatment manual. Families were willing to be recruited and randomised, parents found the format and content of the intervention acceptable, and the attrition rate in the intervention group was low. However, whilst participants were willing to be randomised, there was lower uptake of the UA programme. Participants who attended highlighted how enjoyable and helpful the UA programme and group leaders were, but they would have preferred being allocated to the CUES© group because they were particularly interested in accessing support for their child’s difficulties with uncertainty. To reduce the impact of this in future studies a delayed treatment design could be considered.

Whilst the study was not designed as a fully powered trial, there is preliminary evidence that CUES© led to increased parental self-efficacy, as determined by visual analysis of the CEQ scores and the in-session uncertain situations confidence ratings and the semi-structured interviews. Our findings also provide preliminary evidence of improvements in response to and family impact of an uncertain situation that the children wanted to engage in, based on analysis (using intention to treat) of the uncertain situation vignette ratings at primary endpoint (26 weeks post intervention). In addition, there was reduction in parent reported child anxiety, particularly in relation to separation anxiety, based on data from the Separation Anxiety subscale of the ASC ASD, a parent report measure of child anxiety developed specifically for autistic children. These findings are replicated in the analysis of the Separation Anxiety subscale of the SCARED, a measure not specifically developed for autistic children. Separation anxiety has been commonly reported in young autistic children (Keen et al., 2019). Autistic children may be vulnerable to separation anxiety due to the instrumental support parents provide, such as understanding their child’s needs and advocating for them. This may mean the child relies more on their parent to uncertain navigate situations. By providing the parents and consequently the child with helpful strategies to manage everyday uncertainty, the reliance on parents and consequent anxiety about separation may therefore be reduced. Future work should examine the interaction between IU and separation anxiety in more detail.

## Utility of outcome measures

Many children in the study were willing and able to complete the self-report measures. Parents found the measures acceptable and many commented on how helpful they were to reflect on their child’s behaviour and support needs; however, it could be distressing as parents perceived they should have recognised different facets of anxiety and provided more support for their child. The target behaviour vignette was feasible to use and captures idiographic accounts of uncertain situations that are relevant to the child and family, whilst also providing quantitative data through the change score, as rated by an expert panel.

Between 44% and 66% of questionnaires were not returned, which is in keeping with declining response rates to surveys in the social sciences (National Research Council, 2013). The research team put extensive resource into engaging participants and encouraging responses to follow ups, including sending questionnaires via post with reply-paid envelopes, arranging face-to-face visits/telephone calls to complete questionnaires, offering online completion of questionnaires, sending newsletters with study updates, sending reminders (via email, telephone calls, post, and text) and consulting with a parent advisory committee. These methods were helpful to increase response rates for some but not all participants. It is difficult to find a balance between contacting participants on numerous occasions and providing people with ample opportunity to respond (Harrison et al., 2019). Researchers should consult with community members about what is appropriate and helpful. Parents who did not engage with follow ups did not give reasons, but we expect that participants had many demands on their time. Indeed, previous research has demonstrated that parents of autistic children experience more practical problems, more psychological distress, and lower social support compared to parents who do not have an autistic child (e.g., Picardi et al., 2018). Therefore, we recommend future trials are flexible with data collection methods and anticipate low response rates, particularly in the control arm.

## Strengths and Limitations

CUES© is the first intervention designed for autistic children to help them with uncertainty. Recruitment was achieved through clinical services. However, the results may be biased due to the fact the UA group had high levels of non-uptake and attrition, and we were unable to determine the reasons for this with all families. Those families that did provide a reason indicated that they would have preferred being allocated to the CUES© group because they were particularly interested in accessing support for their child’s difficulties with uncertainty. To reduce the impact of this in future studies, a delayed treatment design could be considered. Finally, the small sample size affects the precision of effect size estimates which means that conclusions cannot be drawn about the effectiveness of CUES©.

## Implications for further research

Our findings provide evidence for the feasibility and acceptability of the CUES© programme. Therefore, we recommend a fully powered randomised controlled trial to provide evidence for the clinical and cost effectiveness of CUES©.

When identifying target uncertain situations, parents spontaneously and most commonly identified school-related situations supporting previous research (Syriopoulou-Delli et al., 2019). This demonstrates the need for interventions to address uncertainty in school settings. Such interventions could improve education outcomes, and quality of life for autistic children and their families.

## Conclusions

The CUES© programme was acceptable to parents and therapists and feasible to implement. Whilst not fully powered, the findings suggest that participants in the CUES© programme experienced increased self-efficacy. Children showed a reduction in anxiety and improvements in responses to uncertain situations. A definitive trial is now required to establish the effectiveness of CUES©. Future research should also explore the interaction between separation anxiety and IU, as well as adaptations to CUES© to make it suitable for the school environment.

## Data Availability

The datasets generated and/or analysed during the current study are available from the corresponding author on reasonable request.

## Declarations

### Trial registration

ISRCTN10139240, 14/05/2018.
